# *catena*-Poly[[diaqua­(2,2′-bipyridine-κ^2^ *N*,*N*′)zinc]-μ-2,2′-[1,4-phenylene­bis(sulfanedi­yl)]diacetato-κ^2^ *O*:*O*′]

**DOI:** 10.1107/S1600536811055310

**Published:** 2012-01-07

**Authors:** Hong Lin, Xiao-Juan Wang

**Affiliations:** a Jinhua Professional Technical College, No. 1188 Wuzhou Street, Jinhua, Zhejiang 321007, People’s Republic of China; b Zhejiang Key Laboratory for Reactive Chemistry on Solid Surfaces, Institute of Physical Chemistry, Zhejiang Normal University, Jinhua, Zhejiang 321004, People’s Republic of China

## Abstract

In the polymeric title complex, [Zn(C_10_H_8_O_4_S_2_)(C_10_H_8_N_2_)(H_2_O)_2_]_*n*_, the Zn^2+^ ion lies on a twofold rotation axis and exhibits an octa­hedral environment, in which it is coordinated by two *trans* O atoms from two symmetry-related 2,2′-[1,4-phenyl­enebis(sulfanedi­yl)]diacetate anions, two N atoms from one 2,2′-bipyridine ligand, and two *cis* O atoms from water mol­ecules. The dihedral angle between the two pyridine rings is 11.5 (1)°. Adjacent Zn^2+^ ions are bridged in a monodentate manner by the diacetate anions, forming a chain structure extending parallel to [101], and are further linked into the final three-dimensional structure by O—H⋯O hydrogen bonds between the coordinating water mol­ecules as donor and the non-coordinating carboxyl­ate O atoms as acceptor atoms.

## Related literature

For background to 1,4-benzene­bis­(thio­acetic acid), including its synthesis and coordination behaviour, see: Yin & Feng (2009 ▶); Yin *et al.* (2009 ▶); Chen *et al.* (2010 ▶); Wang *et al.* (2011**a* ▶,b* ▶); Jiang *et al.* (2012 ▶).
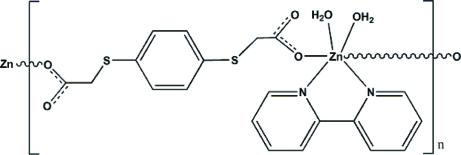


## Experimental

### 

#### Crystal data

[Zn(C_10_H_8_O_4_S_2_)(C_10_H_8_N_2_)(H_2_O)_2_]
*M* *_r_* = 513.87
Monoclinic, 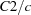

*a* = 20.4396 (8) Å
*b* = 12.8695 (8) Å
*c* = 7.9798 (4) Å
β = 90.765 (3)°
*V* = 2098.88 (19) Å^3^
*Z* = 4
Mo *K*α radiation
μ = 1.41 mm^−1^
*T* = 296 K
0.22 × 0.16 × 0.11 mm

#### Data collection

Bruker APEXII CCD diffractometer
Absorption correction: multi-scan (*SADABS*; Sheldrick, 1996 ▶) *T*_min_ = 0.761, *T*_max_ = 0.853
16253 measured reflections
2448 independent reflections
2237 reflections with *I* > 2σ(*I*)
*R*_int_ = 0.026

#### Refinement

*R*[*F* ^2^ > 2σ(*F* ^2^)] = 0.025
*wR*(*F* ^2^) = 0.070
*S* = 1.04
2448 reflections
147 parameters
3 restraints
H atoms treated by a mixture of independent and constrained refinement
Δρ_max_ = 0.31 e Å^−3^
Δρ_min_ = −0.31 e Å^−3^

### 

Data collection: *APEX2* (Bruker, 2006 ▶); cell refinement: *SAINT* (Bruker, 2006 ▶); data reduction: *SAINT*; program(s) used to solve structure: *SHELXS97* (Sheldrick, 2008 ▶); program(s) used to refine structure: *SHELXL97* (Sheldrick, 2008 ▶); molecular graphics: *SHELXTL* (Sheldrick, 2008 ▶) and *DIAMOND* (Crystal Impact, 2008 ▶); software used to prepare material for publication: *publCIF* (Westrip, 2010 ▶).

## Supplementary Material

Crystal structure: contains datablock(s) I, global. DOI: 10.1107/S1600536811055310/wm2568sup1.cif

Structure factors: contains datablock(s) I. DOI: 10.1107/S1600536811055310/wm2568Isup2.hkl

Additional supplementary materials:  crystallographic information; 3D view; checkCIF report

## Figures and Tables

**Table 1:** Selected bond lengths (Å)

Zn1—O1*W*	2.0896 (11)
Zn1—N1	2.1477 (13)
Zn1—O1	2.1529 (10)

**Table 2:** Hydrogen-bond geometry (Å, °)

*D*—H⋯*A*	*D*—H	H⋯*A*	*D*⋯*A*	*D*—H⋯*A*
O1*W*—H1*WA*⋯O2^i^	0.83 (1)	1.90 (2)	2.7107 (15)	170 (2)
O1*W*—H1*WB*⋯O2	0.82 (1)	1.86 (2)	2.6582 (16)	164 (2)

Symmetry code: (i) 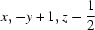.

**Fig. 1.**
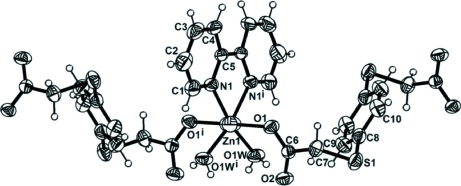
The molecular structure of (I). Displacement ellipsoids are drawn at the 30% probability level. [Symmetry codes: (i) -x + 1,y,-z + 1/2.]

**Fig. 2.**
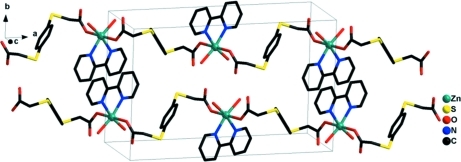
The chain structure of the title complex. All H atoms have been omitted for clarity.

**Fig. 3.**
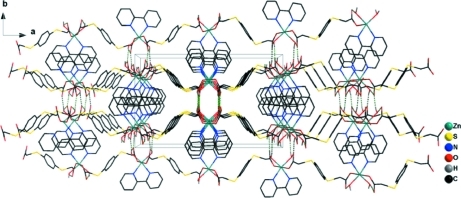
The three-dimensional supramolecular structure built through O—H···O hydrogen bonds (dashed lines).

## supplementary crystallographic information

### Comment

1,4-Benzenebis(thioacetic acid), (C_10_H_10_O_4_S_2_), is a flexible aromatic multi-carboxylate ligand, which can be prepared from 1,4-benzenebisthiol (Yin & Feng, 2009). Compared with rigid ligands, these flexible aromatic carboxylate ligands contain more coordination sites (*viz.* S and O atoms) to construct various extended structures with different metal ions. Recently, some complexes derived from 1,4-benzenebis(thioacetic acid) with bipyridine ligands have been reported (Yin *et al.*, 2009; Chen *et al.*, 2010; Wang *et al.*, 2011**a*,b*; Jiang *et al.*, 2012). Here we report the synthesis and structure of a new complex, [Zn(C_10_H_8_O_4_S_2_)(C_10_H_8_N_2_)(H_2_O)_2_]_n_, (I).

Complex (I) is isotypic with its Co(II) analogue (Jiang *et al.*, 2012). A view on the molecular structure of (I), showing the coordination environment of the Zn^2+^ ion, is presented in Fig. 1. The asymmetric unit consists of one Zn^2+^ ion (situated on a twofold rotation axis), half of a [C_10_H_8_O_4_S_2_]^2-^ anion, half of a 2,2'-bipy molecule, and one coordinating water molecule. The Zn^2+^ ion is six-coordinated by two O atoms from two symmetry-related [C_10_H_8_O_4_S_2_]^2-^ anions (Zn—O 2.1529 (10) Å), two N atoms from one chelating 2,2'-bipy molecule (Zn—N 2.1477 (13) Å), and two water molecules (Zn—O 2.0896 (11) Å) to form a slightly distorted octahedral geometry. The two pyridine rings in the bipy ligand are almost parallel with a dihedral angle of 11.5 (1)°. As shown in Fig. 2, adjacent Zn^2+^ ions are monodentately linked by the [C_10_H_8_O_4_S_2_]^2-^ anions to form a chain structure running parallel to [101]. The chains are further linked by O—H···O hydrogen bonds to form the final three-dimensional supramolecular architecture (Fig. 3).

### Experimental

A mixture of 1,4-benzenebis(thioacetic acid) (0.103 g, 0.4 mmol), ZnCl_2_.6H_2_O (0.054 g, 0.4 mmol), 2,2'-bipy (0.031 g, 0.2 mmol), and Na_2_CO_3_ (0.042 g, 0.4 mmol) in H_2_O (16 ml)/C_2_H_5_OH (2 ml) was placed in a 25 ml Teflon-lined stainless steel vessel and heated at 433 K for 72 h, then cooled to room temperature over 3 d. Colourless crystals suitable for X-ray analysis were obtained.

### Refinement

The carbon-bound H-atoms were positioned geometrically and included in the refinement using a riding model [aromatic C—H 0.93Å and aliphatic C—H 0.97 Å, *U*_iso_(H) = 1.2*U*_eq_(C)]. The oxygen-bound H-atoms were located in difference Fourier maps and refined with an O—H distance restrained to 0.85 Å and *U*_iso_(H) = 1.2*U*_eq_(O).

### Crystal data

**Table:**

[Zn(C_10_H_8_O_4_S_2_)(C_10_H_8_N_2_)(H_2_O)_2_]	*F*(000) = 1056
*M**_r_* = 513.87	*D*_x_ = 1.626 Mg m^−^^3^
Monoclinic, *C*2/*c*	Mo *K*α radiation, λ = 0.71073 Å
Hall symbol: -C 2yc	Cell parameters from 7952 reflections
*a* = 20.4396 (8) Å	θ = 1.9–27.7°
*b* = 12.8695 (8) Å	µ = 1.41 mm^−^^1^
*c* = 7.9798 (4) Å	*T* = 296 K
β = 90.765 (3)°	Block, colourless
*V* = 2098.88 (19) Å^3^	0.22 × 0.16 × 0.11 mm
*Z* = 4	

### Data collection

**Table:**

Bruker APEXII CCD diffractometer	2448 independent reflections
Radiation source: fine-focus sealed tube	2237 reflections with *I* > 2σ(*I*)
graphite	*R*_int_ = 0.026
ω scans	θ_max_ = 27.7°, θ_min_ = 1.9°
Absorption correction: multi-scan (*SADABS*; Sheldrick, 1996)	*h* = −26→26
*T*_min_ = 0.761, *T*_max_ = 0.853	*k* = −15→16
16253 measured reflections	*l* = −10→10

### Refinement

**Table:**

Refinement on *F*^2^	Primary atom site location: structure-invariant direct methods
Least-squares matrix: full	Secondary atom site location: difference Fourier map
*R*[*F*^2^ > 2σ(*F*^2^)] = 0.025	Hydrogen site location: inferred from neighbouring sites
*wR*(*F*^2^) = 0.070	H atoms treated by a mixture of independent and constrained refinement
*S* = 1.04	*w* = 1/[σ^2^(*F*_o_^2^) + (0.043*P*)^2^ + 0.7442*P*] where *P* = (*F*_o_^2^ + 2*F*_c_^2^)/3
2448 reflections	(Δ/σ)_max_ = 0.001
147 parameters	Δρ_max_ = 0.31 e Å^−^^3^
3 restraints	Δρ_min_ = −0.31 e Å^−^^3^

### Special details

**Table:**

Geometry. All e.s.d.'s (except the e.s.d. in the dihedral angle between two l.s. planes) are estimated using the full covariance matrix. The cell e.s.d.'s are taken into account individually in the estimation of e.s.d.'s in distances, angles and torsion angles; correlations between e.s.d.'s in cell parameters are only used when they are defined by crystal symmetry. An approximate (isotropic) treatment of cell e.s.d.'s is used for estimating e.s.d.'s involving l.s. planes.
Refinement. Refinement of *F*^2^ against ALL reflections. The weighted *R*-factor *wR* and goodness of fit *S* are based on *F*^2^, conventional *R*-factors *R* are based on *F*, with *F* set to zero for negative *F*^2^. The threshold expression of *F*^2^ > σ(*F*^2^) is used only for calculating *R*-factors(gt) *etc*. and is not relevant to the choice of reflections for refinement. *R*-factors based on *F*^2^ are statistically about twice as large as those based on *F*, and *R*- factors based on ALL data will be even larger.

### Fractional atomic coordinates and isotropic or equivalent isotropic displacement parameters (Å^2^)

**Table:**

	*x*	*y*	*z*	*U*_iso_*/*U*_eq_	
Zn1	0.5000	0.725191 (16)	0.2500	0.03569 (9)	
S1	0.676748 (19)	0.62114 (4)	0.78141 (5)	0.04863 (12)	
N1	0.44065 (7)	0.85617 (9)	0.31662 (16)	0.0391 (3)	
O1W	0.57320 (6)	0.61941 (8)	0.18683 (14)	0.0435 (3)	
H1WA	0.5703 (10)	0.5693 (13)	0.123 (2)	0.052*	
H1WB	0.5785 (10)	0.5933 (15)	0.2793 (18)	0.052*	
O1	0.54190 (6)	0.72445 (7)	0.49896 (13)	0.0398 (2)	
O2	0.57240 (6)	0.55857 (8)	0.50514 (13)	0.0446 (3)	
C1	0.38250 (9)	0.85086 (14)	0.3913 (2)	0.0484 (4)	
H1A	0.3633	0.7861	0.4061	0.058*	
C2	0.34980 (10)	0.93842 (17)	0.4477 (2)	0.0597 (5)	
H2A	0.3089	0.9329	0.4966	0.072*	
C3	0.37950 (11)	1.03389 (15)	0.4292 (3)	0.0618 (5)	
H3A	0.3594	1.0937	0.4688	0.074*	
C4	0.43876 (10)	1.03996 (13)	0.3523 (2)	0.0532 (4)	
H4A	0.4592	1.1040	0.3391	0.064*	
C5	0.46836 (8)	0.94978 (11)	0.29374 (19)	0.0403 (3)	
C6	0.56711 (6)	0.64669 (10)	0.56961 (17)	0.0319 (3)	
C7	0.59312 (7)	0.66315 (13)	0.74760 (18)	0.0395 (3)	
H7A	0.5652	0.6258	0.8245	0.047*	
H7B	0.5901	0.7365	0.7746	0.047*	
C8	0.71752 (7)	0.69346 (13)	0.6228 (2)	0.0408 (3)	
C9	0.71738 (8)	0.65950 (13)	0.4586 (2)	0.0479 (4)	
H9A	0.6958	0.5983	0.4300	0.057*	
C10	0.75080 (8)	0.78402 (14)	0.6640 (2)	0.0475 (4)	
H10A	0.7518	0.8070	0.7745	0.057*	

### Atomic displacement parameters (Å^2^)

**Table:**

	*U*^11^	*U*^22^	*U*^33^	*U*^12^	*U*^13^	*U*^23^
Zn1	0.04999 (16)	0.02228 (13)	0.03467 (14)	0.000	−0.00485 (10)	0.000
S1	0.0406 (2)	0.0562 (3)	0.0490 (2)	0.00141 (17)	−0.00659 (17)	0.01881 (18)
N1	0.0518 (7)	0.0284 (6)	0.0367 (6)	0.0019 (5)	−0.0129 (5)	−0.0020 (5)
O1W	0.0664 (7)	0.0291 (5)	0.0349 (5)	0.0064 (5)	−0.0039 (5)	−0.0045 (4)
O1	0.0540 (6)	0.0316 (5)	0.0336 (5)	0.0051 (4)	−0.0085 (5)	−0.0008 (4)
O2	0.0661 (7)	0.0290 (5)	0.0385 (5)	0.0007 (5)	−0.0048 (5)	0.0043 (4)
C1	0.0531 (9)	0.0449 (9)	0.0468 (9)	0.0033 (7)	−0.0084 (7)	−0.0017 (7)
C2	0.0590 (11)	0.0643 (12)	0.0555 (11)	0.0169 (9)	−0.0085 (8)	−0.0076 (9)
C3	0.0744 (13)	0.0478 (10)	0.0626 (11)	0.0254 (9)	−0.0234 (9)	−0.0171 (8)
C4	0.0680 (11)	0.0309 (8)	0.0600 (10)	0.0107 (7)	−0.0274 (9)	−0.0082 (7)
C5	0.0546 (8)	0.0258 (6)	0.0398 (7)	0.0028 (6)	−0.0231 (6)	−0.0023 (5)
C6	0.0324 (6)	0.0328 (7)	0.0304 (6)	−0.0034 (5)	0.0012 (5)	0.0042 (5)
C7	0.0400 (7)	0.0479 (8)	0.0307 (7)	0.0045 (6)	−0.0003 (6)	0.0028 (6)
C8	0.0310 (7)	0.0437 (8)	0.0475 (8)	0.0024 (6)	−0.0010 (6)	0.0051 (7)
C9	0.0418 (8)	0.0454 (9)	0.0564 (10)	−0.0066 (7)	0.0025 (7)	−0.0064 (7)
C10	0.0420 (8)	0.0553 (10)	0.0452 (9)	−0.0039 (7)	0.0003 (7)	−0.0075 (7)

### Geometric parameters (Å, °)

**Table:**

Zn1—O1W^i^	2.0896 (11)	C2—C3	1.379 (3)
Zn1—O1W	2.0896 (11)	C2—H2A	0.9300
Zn1—N1^i^	2.1477 (13)	C3—C4	1.367 (3)
Zn1—N1	2.1477 (13)	C3—H3A	0.9300
Zn1—O1^i^	2.1529 (10)	C4—C5	1.392 (2)
Zn1—O1	2.1529 (10)	C4—H4A	0.9300
S1—C8	1.7861 (16)	C5—C5^i^	1.478 (4)
S1—C7	1.8095 (15)	C6—C7	1.5247 (19)
N1—C1	1.339 (2)	C7—H7A	0.9700
N1—C5	1.3447 (19)	C7—H7B	0.9700
O1W—H1WA	0.825 (14)	C8—C9	1.382 (2)
O1W—H1WB	0.817 (14)	C8—C10	1.387 (2)
O1—C6	1.2557 (16)	C9—C10^ii^	1.388 (2)
O2—C6	1.2506 (17)	C9—H9A	0.9300
C1—C2	1.388 (3)	C10—C9^ii^	1.388 (2)
C1—H1A	0.9300	C10—H10A	0.9300
			
O1W^i^—Zn1—O1W	98.69 (7)	C1—C2—H2A	120.8
O1W^i^—Zn1—N1^i^	168.43 (5)	C4—C3—C2	119.51 (17)
O1W—Zn1—N1^i^	92.47 (5)	C4—C3—H3A	120.2
O1W^i^—Zn1—N1	92.47 (5)	C2—C3—H3A	120.2
O1W—Zn1—N1	168.43 (5)	C3—C4—C5	119.63 (17)
N1^i^—Zn1—N1	76.59 (7)	C3—C4—H4A	120.2
O1W^i^—Zn1—O1^i^	86.70 (4)	C5—C4—H4A	120.2
O1W—Zn1—O1^i^	92.97 (4)	N1—C5—C4	121.03 (16)
N1^i^—Zn1—O1^i^	89.66 (4)	N1—C5—C5^i^	115.91 (9)
N1—Zn1—O1^i^	90.74 (4)	C4—C5—C5^i^	123.05 (11)
O1W^i^—Zn1—O1	92.97 (4)	O2—C6—O1	125.11 (13)
O1W—Zn1—O1	86.70 (4)	O2—C6—C7	118.57 (12)
N1^i^—Zn1—O1	90.74 (4)	O1—C6—C7	116.32 (12)
N1—Zn1—O1	89.66 (4)	C6—C7—S1	114.48 (10)
O1^i^—Zn1—O1	179.49 (5)	C6—C7—H7A	108.6
C8—S1—C7	100.80 (7)	S1—C7—H7A	108.6
C1—N1—C5	118.97 (14)	C6—C7—H7B	108.6
C1—N1—Zn1	125.31 (11)	S1—C7—H7B	108.6
C5—N1—Zn1	115.42 (11)	H7A—C7—H7B	107.6
Zn1—O1W—H1WA	127.8 (14)	C9—C8—C10	119.04 (15)
Zn1—O1W—H1WB	97.9 (14)	C9—C8—S1	120.80 (13)
H1WA—O1W—H1WB	104.3 (17)	C10—C8—S1	120.15 (13)
C6—O1—Zn1	125.00 (9)	C8—C9—C10^ii^	120.52 (16)
N1—C1—C2	122.49 (17)	C8—C9—H9A	119.7
N1—C1—H1A	118.8	C10^ii^—C9—H9A	119.7
C2—C1—H1A	118.8	C8—C10—C9^ii^	120.43 (16)
C3—C2—C1	118.3 (2)	C8—C10—H10A	119.8
C3—C2—H2A	120.8	C9^ii^—C10—H10A	119.8
			
O1W^i^—Zn1—N1—C1	7.41 (13)	C2—C3—C4—C5	0.0 (3)
O1W—Zn1—N1—C1	−157.1 (2)	C1—N1—C5—C4	2.5 (2)
N1^i^—Zn1—N1—C1	−176.39 (15)	Zn1—N1—C5—C4	−171.45 (11)
O1^i^—Zn1—N1—C1	94.13 (13)	C1—N1—C5—C5^i^	−178.37 (15)
O1—Zn1—N1—C1	−85.55 (13)	Zn1—N1—C5—C5^i^	7.63 (19)
O1W^i^—Zn1—N1—C5	−179.03 (10)	C3—C4—C5—N1	−2.3 (2)
O1W—Zn1—N1—C5	16.4 (3)	C3—C4—C5—C5^i^	178.72 (18)
N1^i^—Zn1—N1—C5	−2.82 (7)	Zn1—O1—C6—O2	1.0 (2)
O1^i^—Zn1—N1—C5	−92.30 (10)	Zn1—O1—C6—C7	−179.21 (9)
O1—Zn1—N1—C5	88.01 (10)	O2—C6—C7—S1	51.79 (17)
O1W^i^—Zn1—O1—C6	63.39 (12)	O1—C6—C7—S1	−128.01 (12)
O1W—Zn1—O1—C6	−35.15 (12)	C8—S1—C7—C6	55.61 (13)
N1^i^—Zn1—O1—C6	−127.58 (12)	C7—S1—C8—C9	−80.93 (14)
N1—Zn1—O1—C6	155.84 (12)	C7—S1—C8—C10	100.14 (14)
O1^i^—Zn1—O1—C6	14.13 (13)	C10—C8—C9—C10^ii^	−1.0 (3)
C5—N1—C1—C2	−0.6 (2)	S1—C8—C9—C10^ii^	−179.94 (13)
Zn1—N1—C1—C2	172.80 (13)	C9—C8—C10—C9^ii^	1.0 (3)
N1—C1—C2—C3	−1.7 (3)	S1—C8—C10—C9^ii^	179.95 (13)
C1—C2—C3—C4	1.9 (3)		

Symmetry codes: (i) −*x*+1, *y*, −*z*+1/2; (ii) −*x*+3/2, −*y*+3/2, −*z*+1.

### Hydrogen-bond geometry (Å, °)

**Table:**

*D*—H···*A*	*D*—H	H···*A*	*D*···*A*	*D*—H···*A*
O1W—H1WA···O2^iii^	0.83 (1)	1.90 (2)	2.7107 (15)	170 (2)
O1W—H1WB···O2	0.82 (1)	1.86 (2)	2.6582 (16)	164 (2)

Symmetry codes: (iii) *x*, −*y*+1, *z*−1/2.
